# Biomass Related Highly Porous Metal Free Carbon for Gas Storage and Electrocatalytic Applications

**DOI:** 10.3390/ma14133488

**Published:** 2021-06-23

**Authors:** Samantha K. Samaniego Andrade, István Bakos, Gábor Dobos, Attila Farkas, Gábor Kiss, Szilvia Klébert, János Madarász, Krisztina László

**Affiliations:** 1 Department of Physical Chemistry and Materials Science, Budapest University of Technology and Economics, 1521 Budapest, Hungary; ssamaniegoandrade@edu.bme.hu; 2 Research Centre for Natural Sciences, Institute of Materials and Environmental Chemistry, Eötvös Loránd Research Network, Magyar tudósok körútja 2, 1117 Budapest, Hungary; bakos.istvan@ttk.hu (I.B.); klebert.szilvia@ttk.hu (S.K.); 3 Department of Atomic Physics, Budapest University of Technology and Economics, 1521 Budapest, Hungary; dobosg@eik.bme.hu (G.D.); gkiss@mail.bme.hu (G.K.); 4 Department of Organic Chemistry and Technology, Budapest University of Technology and Economics, 1521 Budapest, Hungary; farkas.attila@vbk.bme.hu; 5 Department of Inorganic and Analytical Chemistry, Budapest University of Technology and Economics, 1521 Budapest, Hungary; madarasz.janos@vbk.bme.hu

**Keywords:** N, S co-doped carbon, gas storage, electrocatalysis, oxygen reduction reaction, ORR

## Abstract

In this paper we report the synthesis of a N, S co-doped metal free carbon cryogel obtained from a marine biomass derived precursor using urea as nitrogen source. Natural carrageenan intrinsically contains S and inorganic salt. The latter also serves as an activating agent during the pyrolytic step. The overall 11.6 atomic % surface heteroatom concentration comprises 5% O, 4.6% N and 1% S. The purified and annealed final carbon (CA) has a hierarchical pore structure of micro-, meso- and macropores with an apparent surface area of 1070 m^2^/g. No further treatment was applied. The gas adsorption potential of the samples was probed with H_2_, CO_2_ and CH_4_, while the electrocatalytic properties were tested in an oxygen reduction reaction. The atmospheric CO_2_ and CH_4_ storage capacity at 0 °C in the low pressure range is very similar to that of HKUST-1, with the CO_2_/CH_4_ selectivity below 20 bar, even exceeding that of the MOF, indicating the potential of CA in biogas separation. The electrocatalytic behavior was assessed in an aqueous KOH medium. The observed specific gravimetric capacitance 377 F/g was exceeded only in B, N dual doped and/or graphene doped carbons from among metal free electrode materials. The CA electrode displays almost the same performance as a commercial 20 wt% Pt/C electrode. The oxygen reduction reaction (ORR) exhibits the 4-electron mechanism. The 500-cycle preliminary stability test showed only a slight increase of the surface charge.

## 1. Introduction

A sustainable answer to the continuously growing demand for new carbon sources in energy storage or energy conversion applications lies in the exploitation of the potential of renewable biomass. The estimated carbon content in the overall biomass composition of the biosphere is ≈550 gigatonnes and is distributed among various segments of life [1]. While the lignocellulosic biomass of agricultural origin has been used as a carbon precursor for a long time, the potential of the enormous quantity of marine biomass is far from being fully explored [2]. Carrageenan is a natural polysaccharide extracted from a red seaweed species (Rhodophyceae) with water or aqueous alkali. The global carrageenan industry was valued at USD 762.35 million by 2013 [3]. Regarding its chemical structure, carrageenan is a sulfated polygalactan with a content of 25–30% ester-sulfate groups; it is formed by alternating units of D-galactose and 3,6-anhydro-galactose (3,6-AG) connected by α-1,3 and β-1,4-glycosidic linkage [4]. Commercial carrageenans are sold in the form of stable sodium, potassium, and calcium salts or as a mixture of them. Therefore, the sugar units in the chemical structure and the associated cations jointly determine the properties of the carrageenan [5]. Thus, the various species of carrageenans, like other natural carbon precursors, have the advantage that they contain heteroatoms that are preserved as dopants in the developing carbon matrix. Their inorganic salt content is beneficial during their conversion to highly porous carbon, and plays similar roles as chemical activating agents, e.g., KOH [6]. The catalytic role played by inorganic salts in pyrolysis has been recently discussed by Zhao et al. [7]. Another marine biomass, green algae, was used recently as a precursor for carbon materials with high electrocatalytic activity by Ilnicka et al. [8]. In carbon manufacturing, polymer precursors are especially preferred when carbon with low inorganic impurities is needed. Polyethyleneterephthalate (PET) might be a promising precursor. For its wide commercial application, its conversion to activated carbon also offers a way to reduce the volume of solid polymer waste [9].

The enhanced (electro)catalytic activity of metal free nanostructured carbons is attributed to the presence of heteroatoms (e.g., B, N, O, P, S) and various induced defects. A current review by Zhang et al. summarizes the recent advances in this field, particularly emphasizing the synergistic effects of doping and defects [10]. Sulphur is naturally present in carrageenan and is expected to be saved at least to some extent during its conversion to carbon. Although it has almost the same electronegativity (2.58) as carbon (2.55) the electrocatalytic activity of S-doped graphene was found to be superior to pristine graphene, owing to the electronic spin density of carbon atoms, especially those located on the edges [11,12]. It was shown that in oxygen reduction reactions (ORR) thiophenic sulfur compounds play an important role in withdrawing oxygen from electrolytes and fostering its physical adsorption in small carbon pores. Larger sulfur groups (sulfoxide, sulfones, and sulfonic acids) located in the surface of mesopores favoured access of the electrolyte with dissolved oxygen into the pore system [13,14]. Recently we showed that nitrogen atoms incorporated into carbon materials significantly increase the catalytic and electrocatalytic activity of high surface area carbon materials [15,16,17]. The improved catalytic performance of N-doped carbon materials results from the redistribution of the charge density of adjacent C atoms, due to the electronegativity differences between nitrogen (3.04), and carbon atoms (2.55) (Pauling scale) [18]. The role of nitrogen atoms with different bonding states in the enhanced ORR activity is most often attributed to the graphitic and/or pyridinic nitrogen atoms [19]. N, S co-doped carbon materials have displayed promising electrochemical performances due to the synergistic effect between N and S atoms. Such materials are being investigated for their use in sodium-ion and lithium-ion batteries [20,21], electrocatalysis [22], and supercapacitors [23,24]. Recent DFT calculations revealed that dual S and N doping leads to the redistribution of spin and charge densities and thus to a large number of carbon atom active sites [25].

Besides the advantages of heteroatom doping, de Falco et al. demonstrated the additional benefit of the suitable pore structure in the ORR. The synergy of ultramicropores and hydrophobic surface rich in ether groups and/or electrons enhances the electrocatalytic efficiency of carbon materials and may result in an ORR performance similar to that measured on Pt/C with a 4 electron transfer mechanism [26].

N or/and S doping also enhances the adsorption performance for H_2_, CH_4_, and CO_2_ [27]. They concluded that hydrogen adsorption is mainly related to the specific surface area, while the adsorption of CO_2_ is significantly influenced by sulfur doping. N doping is particularly efficient in promoting the uptake of CO_2_ [28]. It was reported recently that O, N, S doped carbon (7.19 at% O, 4.15 at% N and 1.01 at% S) may adsorb more hydrogen gas at −196 °C than most reported porous carbons, covalent organic frameworks (COFs) or metal organic frameworks (MOFs) [29]. The same authors also pointed out that the sulfonyl group is the one with the highest potential to adsorb H_2_.

In this paper we report the synthesis of a N, S co-doped carbon aerogel obtained from marine biomass. While S is intrinsic in carrageenan, urea was added as a nitrogen source. The application potential of this carbon was tested in electrocatalysis and in the adsorption of environmentally relevant gases.

## 2. Materials and Methods

### 2.1. Synthesis

**Figure 1:**
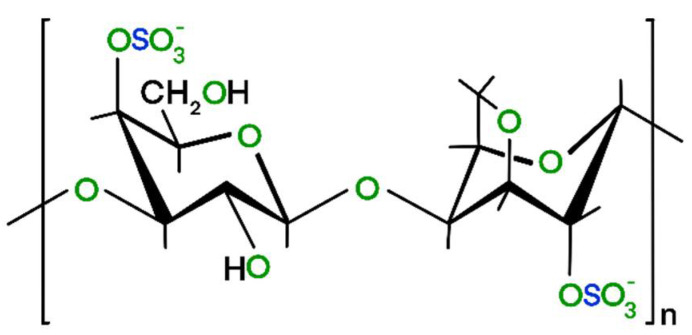
The chemical structure of a monomer unit of ι-carrageenan.

ι-carrageenan powder and urea beads (98%) were bought from Sigma-Aldrich (Budapest, Hungary). The commercial grade, Type II ι-carrageenan precursor contained 4–6% potassium, 2–4% calcium, and 1–2% sodium. The cryogel samples were synthetized according to the method of Li et al. [30]. Briefly, 4 g urea was dissolved in 200 mL of distilled water and warmed to 80 °C. 4 g of ι-carrageenan was added to the solution and stirred for 1 h. ι-carrageenan contains about 28 to 30% ester sulfate and its 3,6-anhydro-galactose content is 25–30% (Figure 1). Due to its strongly anionic half-ester sulfate groups it readily formed a hydrogel in the presence of the potassium and calcium ions. After freeze drying, the dry polymer aerogel (PA) was obtained with a yield of 93%.

PA was first carbonized in a rotary quartz reactor at 700 °C in a dry N_2_ atmosphere, providing a raw carbon aerogel (CR) with a yield of 10%. After removing the inorganic impurities with aqueous 1.0 M HCl (yield 53%), the washed sample (CW) was annealed in argon flow at 1000 °C for 1 h, resulting in the annealed carbon sample (CA) with an overall yield of 2.4%.

### 2.2. Characterization Methods

Low temperature (−196.15 °C) nitrogen adsorption measurements were performed after 24 h degassing at 110 °C on a NOVA 2000e (Quantachrome, Boynton Beach, FL, USA) automatic volumetric instrument. The apparent surface area *S_BET_* was determined using the Brunauer–Emmett–Teller (BET) model [31]. A pore volume *V*_0.98_ was estimated from the amount of vapor adsorbed at *p*/*p*_0_ = 0.98, assuming that the adsorbed gas was present as liquid N_2_. The Dubinin–Radushkevich (DR) plot [32] was used to calculate the micropore volume *V_micro_*. The pore size distribution in the micro and mesopore regions was computed by Quenched Solid Density Functional Theory (QSDFT) [33]. Evaluation of the primary adsorption data was performed with the Quantachrome ASiQwin software (version 3.0). Scanning electron microscopic (SEM) images of the gold coated samples were taken by a JEOL JSM 6380LA (Jeol Ltd., Tokyo, Japan). Elemental mapping was performed by the energy dispersive X-ray spectroscopy (EDS) option of the same instrument. 

Powder X-ray diffractograms (XRD) were obtained in the range 2*θ* = 10–130° with an X’Pert Pro MPD (PANalytical Bv., Almelo, The Netherlands) X-ray diffractometer using an X’celerator type detector and monochromatic Cu Kα radiation with a Ni filter foil (λ = 1.5406 Å) at 40 keV and 30 mA.

Raman spectra were obtained using a LabRAM (Horiba Jobin Yvon) instrument. The laser source was a λ = 532 nm Nd-YAG (laser power at the focal point was 15 mW). A 0.6 OD filter was used to reduce the power of the beam. Parameter optimization and data analysis were performed with LabSpec 5 software. Fourier-transform infrared (FTIR) spectra were collected on an attenuated total reflection Fourier-transform infrared Tensor 37 spectrophotometer (ATR-FTIR, Bruker) with a Specac Golden Gate ATR unit, and are shown here after background correction.

The surface chemical composition was studied by X-ray photoelectron spectra (XPS) using a Thermo Fisher XR4 twin anode X-ray source (Thermo Scientific, Paisley, UK) and a Specs Phoibos 150 hemispherical electron energy analyzer with an MCD9 detector (SPECS Surface Nano Analysis GmbH, Berlin, Germany). The MgKα radiation employed (1253.6 eV) was not monochromated. A Gaussian-Lorentzian function mixed set was used to fit the peaks on each spectrum after subtracting Shirley-type backgrounds using CasaXPS.

The electrocatalytic tests were performed using a glassy carbon (GC) rotating disc electrode (RDE, Pine Research Instrumentation, Durham, NC, USA) and a rotating ring-disc electrode (RRDE) (GC disc and Pt ring). The ink for the working electrodes was prepared by dispersing 2 mg powdered carbon (CA) in a mixture of 1.6 mL MilliQ water, 0.4 mL isopropyl alcohol and 8 µL 5% Nafion^®^ solution. After 30 min sonication the ink was pipetted onto the dry mirror-polished GC and dried at room temperature. The loading was 100 μg/cm^2^. Measurements were implemented in 0.1 M KOH electrolyte using three-electrode systems with a hydrogen electrode as reference and a Pt wire as the counter electrode in a three-compartment PFTE cell (Figure S1). All potentials are given in the Reversible Hydrogen Electrode (RHE) scale. A detailed description is given in reference [16].

Carbon dioxide and methane isotherms were measured at 0 °C up to atmospheric pressure with an AUTOSORB-1 (Quantachrome, Boynton Beach, FL, USA) computer-controlled analyzer. An Autosorb 1C (Quantachrome, Boynton Beach, FL, USA) static volumetric instrument was used to perform hydrogen sorption experiments with high purity hydrogen (99.999%) at −196 °C.

## 3. Results and Discussion

### 3.1. Development of the Morphology during the Synthesis

**Figure 2:**
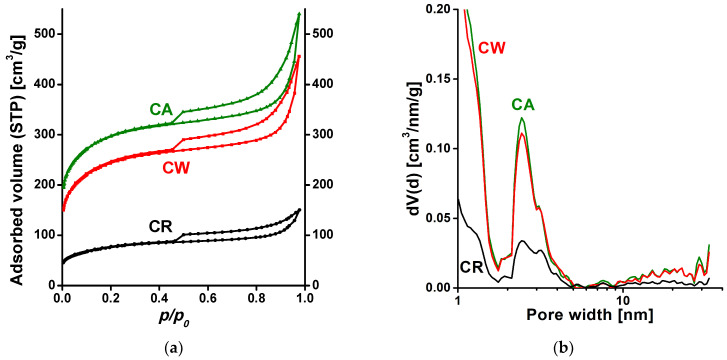
(**a**) Low temperature N_2_ adsorption/desorption isotherms; (**b**) Pore size distribution functions were estimated by quenched solid density functional theory (QSDFT) (Kernel: N_2_ at 77 K on carbon, slit/cylindrical pores, adsorption branch).

Low temperature (−196 °C) nitrogen adsorption isotherms of the carbon samples at different stages of the synthesis are presented in Figure 2a. According to the latest IUPAC classification, the three isotherms are of composite Type IV +Type II with a H4 hysteresis loop with the expected sharp step-down at *p*/*p*_0_ = 0.45 [34].

The shape and the type of the hysteresis loop imply that the samples contain micro-, meso- and macropores that form an interconnected network. As the macropores are not completely filled with pore condensate, the total pore volume of the samples cannot be deduced from the nitrogen adsorption isotherms, instead, we estimated the liquid equivalent *V*_0.98_ of the gas adsorbed at *p*/*p*_0_ = 0.98. The development of the carbon texture during the synthesis steps is simultaneously revealed by the upward shift of the three isotherms, the pore size distribution curves (Figure 2b) and the XRD signals shown in the Supplementary Material Figure S2. The enhancement between CR and CW is related to the removal of inorganic minerals that survive the pyrolysis step, while the moderate shift from CW to CA results from the consecutive thermal treatment. The data deduced from the isotherms are shown in Table 1.

**Figure 3:**
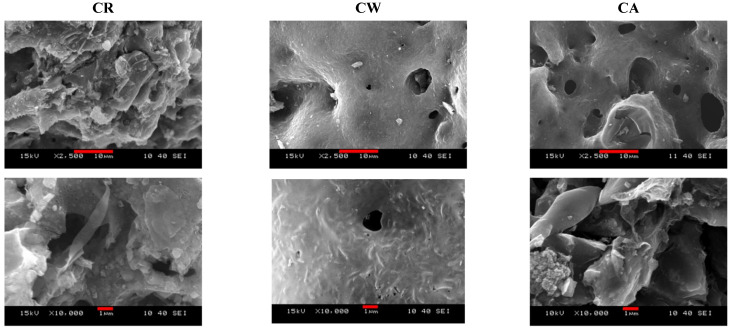
SEM images of carbon aerogel samples CR, CW, and CA. The scale bars are 10 and 1 μm in the upper and lower raw, respectively.

The corresponding images in Figure 3 also confirm the changing morphology. The elemental mapping and apparent bulk composition of the three carbon samples (Figure S3, Table S1) indicate that K and Ca salts were removed during the acidic washing. The removal of the inorganic impurities and the development of the final CA texture can also be tracked from the Raman and FTIR spectra (Figure S4).

**Figure 4:**
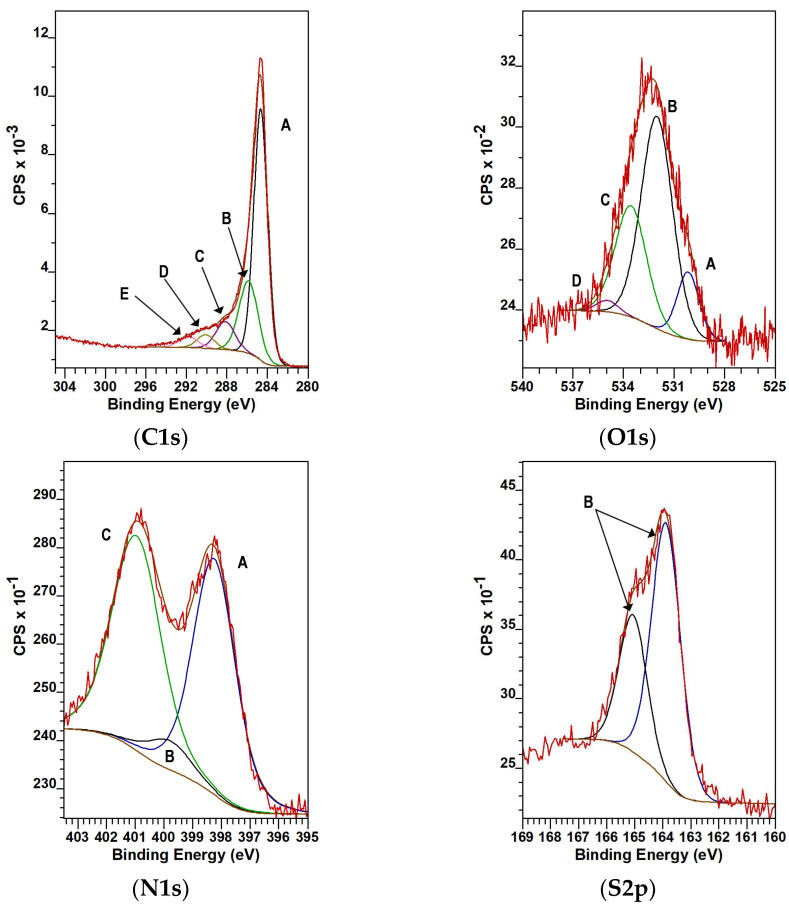
Deconvoluted C1s, O1s, N1s and S2s regions of the CA sample.

The C1s, O1s, N1s and S2s regions of the XPS spectrum of the CA sample are shown in Figure 4. (Figure S5 shows the corresponding regions of the intermediate samples.) Although EDS is intrinsically less sensitive to low atomic number elements, the discrepancy between the EDS and XPS data (Table 2) indicates an inhomogeneous distribution of the nitrogen and sulfur. The concentrations of the metal impurities, Na, K and Ca, are below the detection limit of XPS, thus confirming the observations of the above mentioned methods.

The total O + N + S heteroatom/carbon ratio on the surface of the purified sample is close to 11%, with practically an equal share of the O and N species. The species revealed by the deconvolution of the C1s, O1s, N1s and S2p regions were assigned according to Table S2. Based on the C1s and O1s regions, the surface of the CA carbon is decorated with carbonyl, epoxy, and carboxylic groups. Nitrogen in three different forms, namely in pyridinic, pyrrolic and quaternary, was detected. The S2p peak is split into 2p3/2 and 2p1/2 with 2:1 intensity and a 1.2 eV binding energy gap. The double peak at around 163.8 eV, 165 eV shows the exclusive presence of thiophenic compounds in the final product.

The brief conclusion is that the purified CA carbon has a surface area of 1070 m^2^/g and a total pore volume of 0.83 cm^3^/g. It contains O, N and S heteroatoms at 4.6, 5.0 and 1.0 atomic %, respectively. Based on these textural and surface chemical features this carbon is a promising candidate for gas adsorption and electrocatalysis applications. Without further optimization the gas storage performance was probed with CO_2_, H_2_ and methane, while the electrocatalytic properties of the CA carbon were tested in an oxygen reduction reaction.

### 3.2. Gas Storage Application

**Figure 5:**
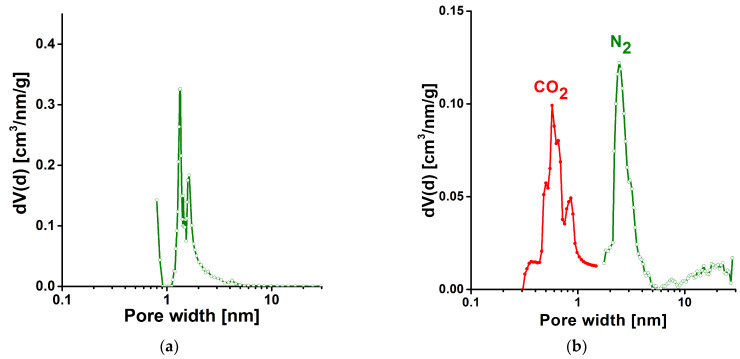
Pore size distribution of samples tested for gas storage. (**a**) HKUST-1, calculated with Barret–Joyner–Halenda (BJH) model [38]; (**b**) CA, derived from CO_2_ and N_2_ isotherms, calculated with QSDFT. Kernel: slit/cylindrical pores, adsorption branch.

H_2_ and CH_4_ were selected as they have considerably higher energy per unit mass in fuel application. Nevertheless, their low density at ambient temperature results in a poor energy per unit volume ratio. By enhancing the latter with adsorption-based gas storage technology could however open an avenue to them in portable power applications. At the same time, sorption related gas storage could offer a solution for reducing CO_2_ emission. The capture of CO_2_ produced in combustion processes and its storage could be an alternative to the present amine absorption process, which is hampered by several technical problems. Adsorption of CO_2_ by highly porous materials also could allow its large scale separation by the well-known pressure or temperature swing adsorption technologies [35]. As metal organic frameworks (MOFs) are among the materials with the greatest potential for sorption-based gas storage applications, copper benzene-1,3,5-tricarboxylate (HKUST-1) synthesized in our laboratory was used as a comparison [36]. The 3D network of HKUST-1 incorporates pores of three well defined sizes of 0.5, 1.1 and 1.35 nm [37,38]. The detailed characterization of the HKUST-1 used for comparison was presented in our previous work [39]. The almost exclusively microporous MOF sample was obtained from a solvothermal synthesis performed in an ethanol–water mixture. The apparent surface area *S_BET_* and total pore volume are 1500 m^2^/g and 0.62 cm^3^/g, respectively [39]. The former exceeds, while the latter remains below the corresponding values of CA (Table 1). As no kernel files necessary for DFT-based calculations are available, the pore size distribution of HKUST-1 was determined with the Barret–Joyner–Halenda (BJH) model [40]. However, this method underestimates the pore size of mesopores narrower than ~10 nm by ca. 20–30% [34]. Nevertheless, the estimated distribution curve in Figure 5a clearly reflects the dual size distribution of this MOF in the supermicropore range, while its smallest pore size lies much beyond the limits of the method.

Figure 5b combines the pore size distribution curves of the CA sample obtained from CO_2_ and N_2_ isotherms with the QSDFT calculations. The curve computed from nitrogen data confirms the mesoporous character of CA, as anticipated from the shape of the isotherm. In using CO_2_ as one of the test gases in this work, we take advantage of the fact that CO_2_ is also a frequently used gas to probe the pore size distribution of carbon materials. The higher kinetic energy of CO_2_ at the temperature of the isotherm measurements allows pores to be revealed that are inaccessible to nitrogen. Figure 5b clearly shows that CA also possesses pores in the ultramicropore range. 

**Figure 6:**
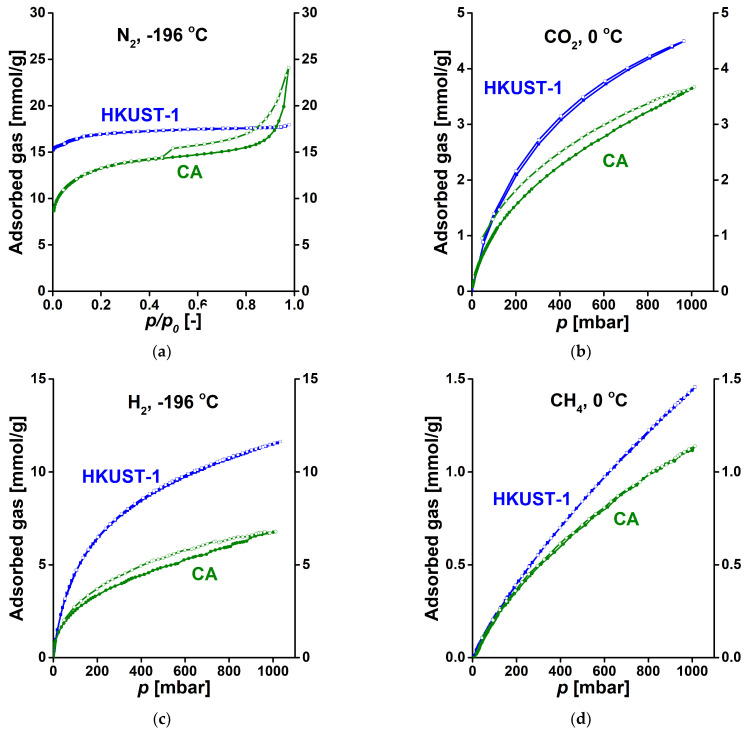
Comparison of adsorption isotherms of CA and HKUST-1 measured with (**a**) nitrogen, (**b**) carbon dioxide; (**c**) hydrogen; (**d**) methane.

Figure 6 shows the hydrogen, CH_4_ and CO_2_ adsorption isotherms of the CA samples up to atmospheric pressure. A recent publication reporting a new synthesis route of cellulose-based carbons has compared the atmospheric CO_2_ adsorption of several cellulose-based materials at 0 °C, 1 bar. The 3.64 mmol/g capacity of the CA sample falls into the 3.40–6.75 mmol/g range reported [6]. 

While all the adsorption isotherms are practically reversible in HKUST-1, only CH_4_ shows a reversible adsorption on the CA. All the test gases, i.e., hydrogen, CH_4_ and CO_2_ exhibit a higher uptake on the MOF, but the deviation is more limited in case of the isotherms measured at 0 °C. In high pressure applications the higher total pore volume of CA can lead to an improved performance. 

The CO_2_ uptake of both porous materials is about four times higher than that of methane measured under the same conditions. This observation can be utilized in gas separation. As in Kamran et al. [6] the ideal adsorbed solution theory (IAST) [41] was applied to estimate and compare the CO_2_/CH_4_ selectivity of the samples. The initial sections of the isotherms were fitted to the linear Henry model. The full CO_2_ and CH_4_ adsorption isotherms of each material also gave a reasonable fit to the single-site Langmuir–Freundlich model [42,43]

(1) $$n_{ads}=\frac{n_{sat}Kp^{m}}{1+Kp^{m}}$$

where *n_ads_* is the quantity adsorbed at equilibrium pressure *p*, *n_sat_* is the saturation capacity, *K* is the equilibrium constant of the Langmuir model and *m* (>1) is the Freundlich exponent. The Henry constants *K_H_* for both gases and the calculated initial selectivity, as well as the fitted Langmuir–Freundlich parameters, are listed in Table 3.

**Figure 7:**
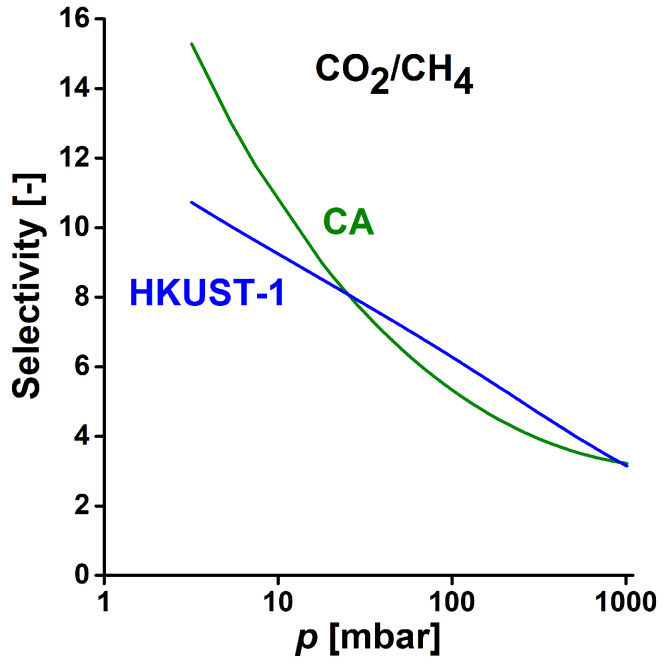
Comparison of the CO_2_/CH_4_ selectivity of CA and HKUST-1 from IAST using the Langmuir–Freundlich fit of the isotherms.

The selectivity for the CO_2_/CH_4_ gas mixture, calculated as

(2) $$S=\frac{n_{CO_{2}}\;\cdot \;p_{CH_{4}}}{n_{CH_{4}}\;\cdot \;p_{CO_{2}}}$$

is shown in Figure 7. A semi-logarithmic pressure scale is used for clearer comparison.

The selectivity of HKUST-1 in this representation displays a linear decrease. Increasing pressure also reduces the selectivity of CA monotonically, but in a more complex way. Below ca 20 bar the estimated selectivity of the carbon significantly exceeds that of the HKUST-1, then up to the upper limit of the pressure range investigated the MOF performs slightly better. That is in good agreement with the ratio of the Henry constants. This finding indicates that CA has potential applications in the separation of CO_2_ and methane from biogas.

### 3.3. Electrocatalytic Application

**Figure 8:**
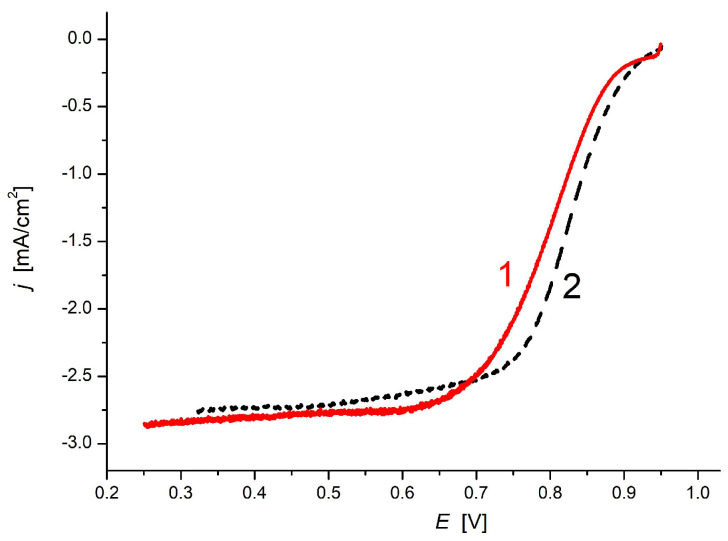
Linear potential sweep of CA sample (solid red line) and a 20 wt% Pt/C electrode (dashed black line) measured in O_2_ saturated 0.1 M KOH solution with a rotating disk electrode (1225 rpm). Loadings: 100 µg/cm^2^, Sweep rate: 5 mV/s.

The potential of CA as an electrode in the ORR and its electrochemical characteristics were investigated. The powdered electrode material was tested in a three-electrode cell configuration in O_2_ saturated 0.1 M KOH electrolyte. Figure 8 compares the results of the RDE measurements in the O_2_ saturated 0.1 M KOH on the 100 µg/cm^2^ CA and a 20 wt% Pt/C commercial electrode (Quintech): the performance of the CA electrode is similar to that of the Pt/C electrode.

**Figure 9:**
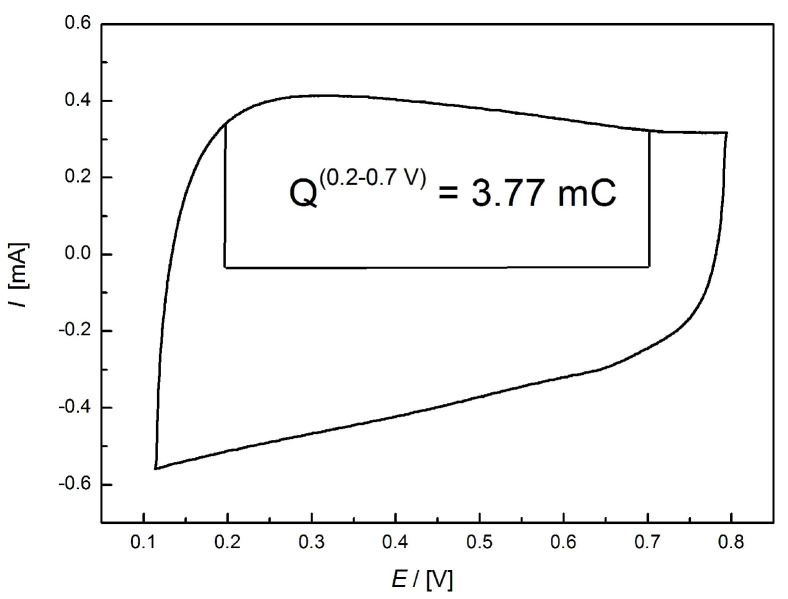
Cyclic voltammogram of the CA sample after ORR measurements. (Loadings: 100 µg/cm^2^, Sweep rate: 50 mV/s.).

According to the results of the cyclic voltammetric measurements the specific gravimetric capacitance *Cg* of the CA electrode in 0.1 M KOH was approximately 377 F/g. This value was estimated from the anodic charge measured between 0.2 and 0.7 V (Figure 9). The 3.77 mC corresponds to an electrode of geometrical surface area of 0.19625 mm^2^ loaded with 20 µg catalyst, i.e., 100 µg/cm^2^ loading. These data indicate that the mass specific capacitance is roughly 377 F/g. This value is higher than the specific capacitance of several high performance electrode materials [44,45] as shown in Table 4. Comparison of the gravimetric capacitances listed in Table 3 leads us to the conclusion that practically only doped graphene and B containing carbons display a higher value than our biomass-based CA carbon. Note that carbon dots with double metal doping may exhibit much higher capacitance [46]. 

**Figure 10:**
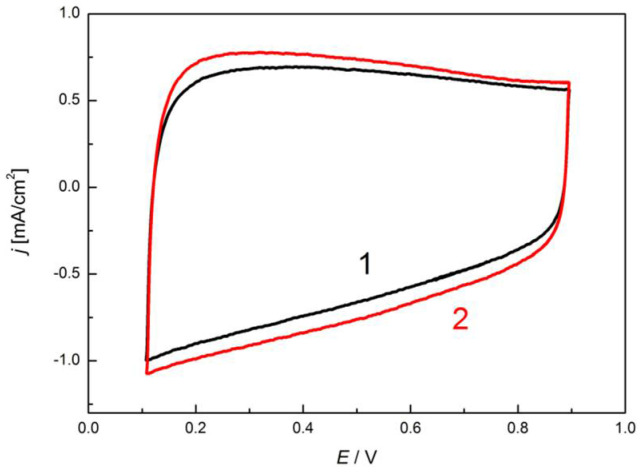
Cyclic voltammogram of a freshly prepared electrode in 0.1 M KOH (1), and after 500 cycles between potential limits 0.1 and 0.9 V (2).

A 500-cycle preliminary stability test was carried out by cyclic polarization of a freshly prepared electrode in the potential range between 0.1 and 0.9 V relevant to ORR. After the 500 cycles only a slight increase of the surface (charge) was observed, as demonstrated in Figure 10.

**Figure 11:**
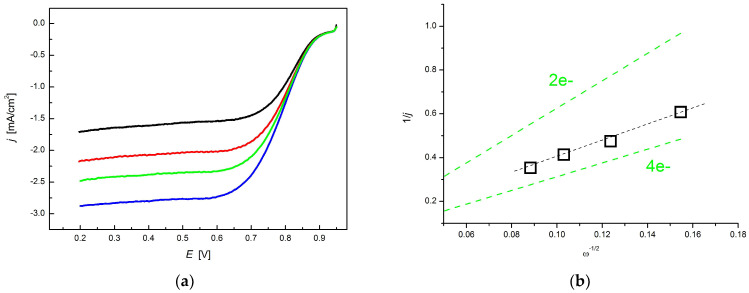
(**a**) Linear sweep voltammograms of CA sample (100 µg/cm^2^) measured in O_2_-saturated 0.1 M KOH solution with a rotating disk electrode (RDE). Rotation rates: 400 (black), 625 (red), 900 (green), 1225 rpm (blue). Sweep rate: 5 mV/s; (**b**) Koutecky–Levich plot from the linear sweep voltammetry responses at 0.30 V. The theoretical KL slopes of the 2e^−^ and 4e^−^ routes are shown for comparison.

The electrocatalytic properties were examined with a rotating disc electrode (RDE) in 0.1 M KOH electrolyte. The polarization curves at different potentials and rotation rates (400–1225 rpm) are shown in Figure 11a. The Koutecky–Levich (KL) equation was used to describe the correlation between the current densities and rotation rate

(3) $$\frac{1}{j}=\frac{1}{j_{k}}+\frac{1}{j_{lim}}=\frac{1}{j_{k}}+\frac{1}{0.62nFCD^{\frac{2}{3}}\nu^{-\frac{1}{6}}\omega^{1/2}}$$

where *j* is the current density, *j_k_* is the kinetic current density, *j_lim_* is the limiting diffusion current density, *n* is the number of electrons transferred in ORR per oxygen molecule, *F* is the Faraday constant, *D* is the diffusion coefficient of oxygen in the electrolyte, *ν* is the kinematic viscosity of the electrolyte, *C* is the concentration of oxygen in the electrolyte, and *w* is the rotation rate in rad/s [66].

The oxygen reduction reaction in aqueous media occurs mainly through two different pathways. The four-electron reduction pathway transforms O_2_ directly to H_2_O. The other route is a two-electron pathway through peroxide formation [67]. In polymer electrolyte membrane fuel cells (PEMFCs) the preferred ORR pathway is a four-electron transfer. Depending on the pH of the reaction media the 4e^−^ reduction route has different thermodynamic potentials ranging from 0.401 V in alkaline media (Equation (4)) to 1.230 V in acidic media (Equation (5)) [67,68]:

(4) $$O_{2}+2H_{2}O+4e^{-}\;\to 4OH^{-}\;(0.401\;V\;\mathrm{vs}.\;\mathrm{standard}\;\mathrm{hydrogen}\;\mathrm{electrode},\;\mathrm{SHE})$$

(5) $$O_{2}+4H^{+}+4e^{-}\;\to 2H_{2}O\;\left(1.229\;V\;\mathrm{vs}.\;\mathrm{SHE}\right)$$

The 2e^−^ reduction follows a two-step mechanism. In basic media, the first step is shown in Equation (6). There are two possible reactions for the second step: a two-electron reduction of HO_2_^−^ (Equation (7)) or its chemical disproportionation (Equation (8)) [68]:

(6) $$O_{2}+{\;H}_{2}O+2e^{-}\;\to {\mathrm{HO}}_{2}^{-}\;\left(-0.076\;V\;\mathrm{vs}.\;\mathrm{SHE}\right)$$

(7) $${\mathrm{HO}}_{2}^{-}+{\;H}_{2}O+2e^{-}\;\to 3{\mathrm{HO}}^{-}\;\left(0.878\;V\;\mathrm{vs}.\;\mathrm{SHE}\right)$$

(8) $$2{\mathrm{HO}}_{2}^{-}\;\to 2{\mathrm{HO}}^{-}+O_{2}$$

In acidic media, the 2e^−^ mechanism for ORR is shown by Equations (9) and (10):

(9) $$O_{2}+2H^{+}+2e^{-}\;\to 2H_{2}O_{2}\;\left(0.695\;V\;\mathrm{vs}.\;\mathrm{SHE}\right)$$

(10) $$H_{2}O_{2}+2H^{+}+2e^{-}\;\to 2H_{2}O\;\left(1.776\;V\;\mathrm{vs}.\;\mathrm{SHE}\right)$$

The pore size distribution curves in Figure 5b reveal that CA also possesses pores in the ultramicropore range. As has been recently pointed out, ultramicropores work as pseudocatalytic centers for ORR by promoting strong O_2_ adsorption. The thus facilitated O=O bond splitting orientates the ORR through a 4e^−^ reduction mechanism [26]. Figure 11b implies that in case of CA the 4e^−^ route is indeed the dominant pathway.

## 4. Conclusions

A highly porous nanostructured carbon material was obtained from a marine biomass—urea physical hydrogel. While S is intrinsically contained in the carrageenan, urea was added as a nitrogen source during the synthesis process, yielding a N, S double doped carbon material after pyrolysis. Aqueous 1.0 M HCl washing successfully removed the inorganic minerals of the carrageenan precursor that served also as an activating agent during the pyrolysis, thus giving access to fully or partially plugged voids. The final porous texture developed after high temperature annealing. The resulting CA carbon possesses a hierarchical pore system of micro-, meso- and macropores with an apparent surface area of 1070 m^2^/g. XPS measurements showed that the total O, N and S content of the final CA carbon was 4.6, 5.0 and 1.0 at%, respectively, i.e., the heteroatom/carbon ratio was close to 11%. The metal content was below the detection limit. While the H_2_ adsorption performance tested at −196 °C was exceeded by HKUST-1, the atmospheric CO_2_ and CH_4_ storage capacities at 0 °C in the low pressure range were very similar to those of the MOF. The CO_2_/CH_4_ selectivity below 20 bar was even better in the CA, implying potential applications in biogas separation. In aqueous KOH medium the CA electrode exhibited almost the same performance as the commercial platinum loaded electrode used for comparison. The electrode displayed the 4-electron ORR mechanism and a promising stability in a 500-cycle preliminary test. On considering only metal free electrode materials, the specific gravimetric capacitance 377 F/g of CA was exceeded only by B, N dual doped and/or graphene doped carbons. 

## Figures and Tables

**Table 1:** Porous characteristics of carbon aerogel samples ^1^.

Sample	*S_BET_*	*V*_0.98_	*V_micro_*	
	m^2^/g	cm^3^/g		%
CR	272	0.23	0.10	42
CW	871	0.70	0.32	46
CA	1070	0.83	0.40	48

^1^ Apparent surface area from BET model, *V*_0.98_ is the liquid equivalent of the gas adsorbed at *p*/*p*_0_ = 0.98, and *V_micro_* refers to the micropore volume. *V_micro_* [%] estimates the contribution of the adsorbed N_2_ filling the micropores.

**Table 2:** Surface compositions (atomic %) measured by XPS.

Sample	C	O	N	S	K	Ca	Na
CR	67.2	20.5	5.9	0.5	2.1	3.2	0.6
CW	81.9	6.9	9.3	2.0	n.d. *	n.d. *	n.d. *
CA	89.4	4.6	5.0	1.0	n.d. *	n.d. *	n.d. *

* n.d.: not detected.

**Table 3:** Parameters derived from Henry and Langmuir–Freundlich fits.

Sample	Gas	Henry			Langmuir–Freundlich			
		*K_H_*	R	$\frac{K_{H,CO_{2}}}{K_{H,\;CH_{4}}}$	*n_sat_*	*K*	*m*	R
		$\frac{mmol}{g\cdot mbar}$			$\frac{mmol}{g}$	$\frac{1}{mbar}$		
CA	CO_2_	0.463	0.9974	10.5	12.5	0.00424	0.660	0.99987
	CH_4_	0.044	0.9963		2.75	0.00088	0.963	0.99953
HKUST-1	CO_2_	0.232	0.9998	5.7	8.30	0.00485	0.799	0.99999
	CH_4_	0.041	0.9984		7.01	0.00046	0.915	0.99997

**Table 4:** Gravimetric capacitance of dual doped metal free carbon materials in KOH electrolyte.

Electrode Material	*C_g_*	Reference
	F/g	
P/N co-doped ordered mesoporous carbon	210	[47]
N, P co-doped graphene	219	[48]
B, N co-doped graphene	225	[49]
N, S co-doped graphene	264.3	[50]
N, P dual-doped hierarchically porous carbon	289	[51]
N, S co-doped flexible graphene paper	305	[52]
N, S co-doped graphene oxide	307	[53]
3D N, S co-doped graphene hydrogel	320	[54]
N, S co-doped carbon	322	[55]
N, S co-doped porous carbon from ionic liquid precursor	347	[56]
N, S co-doped templated porous carbon	367	[57]
**N, S co-doped biomass-based carbon aerogel (CA)**	**377**	**This work**
N,S co-doped porous graphitic carbon from lotus leaves	385	[58]
B, N co-doped porous carbon foam	402	[59]
N, S co-doped graphene-enhanced hierarchical porous carbon foam	405	[60]
N, P co-doped high performance 3D graphene	413	[61]
N, S co-doped carbon (based on polymer from N and S containing aromatic precursors)	461.5	[62]
N, S co-doped graphene material	503	[63]
Vertically-aligned BC2N nanotube arrays	547	[64]
N, S co-doped graphene	566	[65]

## Data Availability

Not applicable.

## Supplementary Materials

The following are available online at https://www.mdpi.com/article/10.3390/ma14133488/s1, Figure S1: Figure S2: XRD diffractograms of the carbon aerogel samples. Figure S3: Elemental mapping from SEM-EDS analysis. Figure S4: (a) Baseline corrected and normalized Raman spectra. (b) FTIR spectra of the precursors and CA. Figure S5: Deconvoluted regions of the XPS spectra of the porous carbon intermediates. Table S1: Elemental compositions (atomic %) measured by EDS. Table S2: Assignation of the chemical states in the C1s, O1s, N1s, and S2p 3/2 regions.
