# Supplementary material for: Biomass Related Highly Porous Metal Free Carbon for Gas Storage and Electrocatalytic Applications
Source: Materials (Basel). 2021 Jun 23;14(13):3488. doi: 10.3390/ma14133488 (PMC8269557; doi:10.3390/ma14133488)
Supplement: Supplementary file 1 [file materials-14-03488-s001.zip › materials-1245511-supplementary.pdf]

**SUPPLEMENTARY MATERIAL**

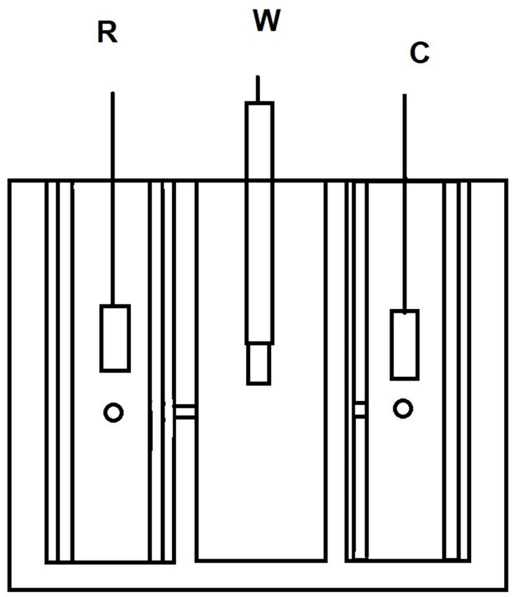


**Figure S1**. Scheme of the PFTE cell used in the electrochemical measurements.
R: reference electrode, W: working electrode, C: counter electrode

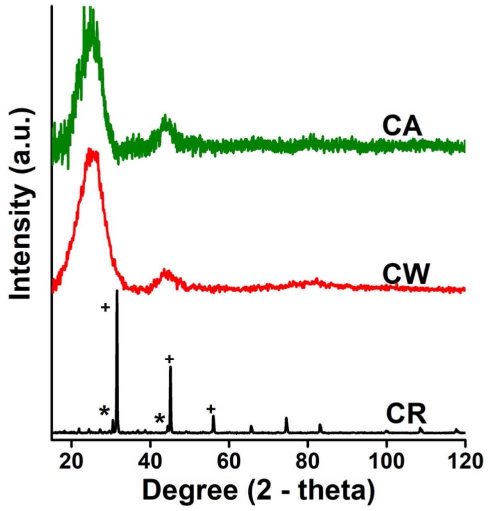


**Figure S2.** XRD diffractograms of the carbon aerogel samples. Peaks labeled with + and * correspond to calcium sulfide (CaS) and potassium sodium sulfate ($KNaSO_4$), respectively.

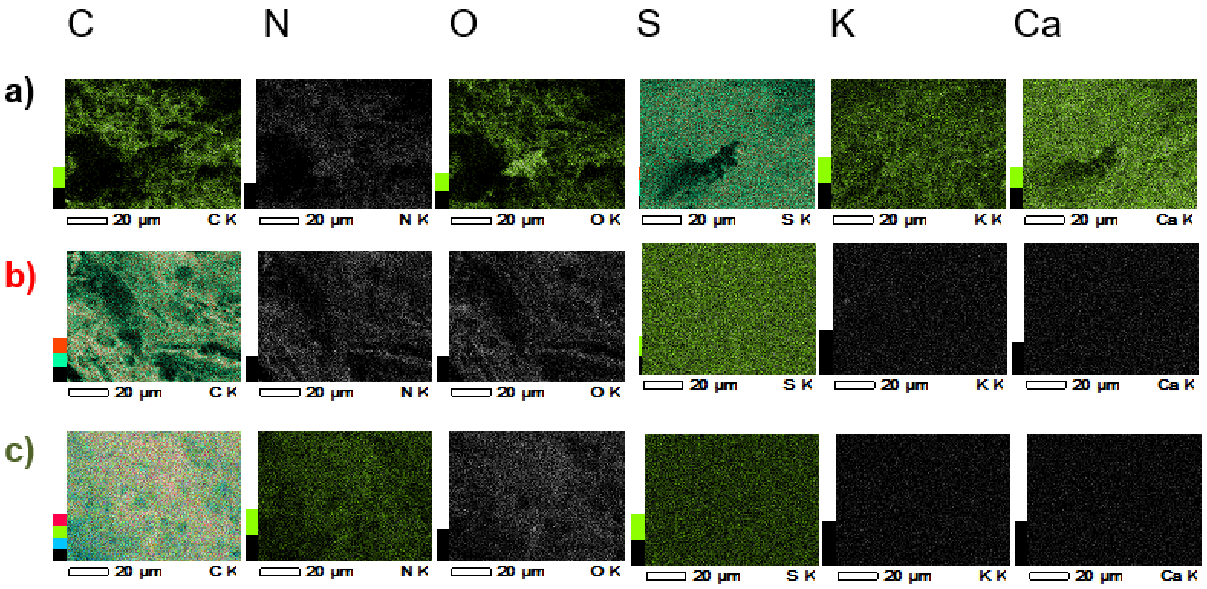


**Figure S3.** Elemental mapping from SEM-EDS analysis. (**a**) CR; (**b**) CW; (**c**) CA.

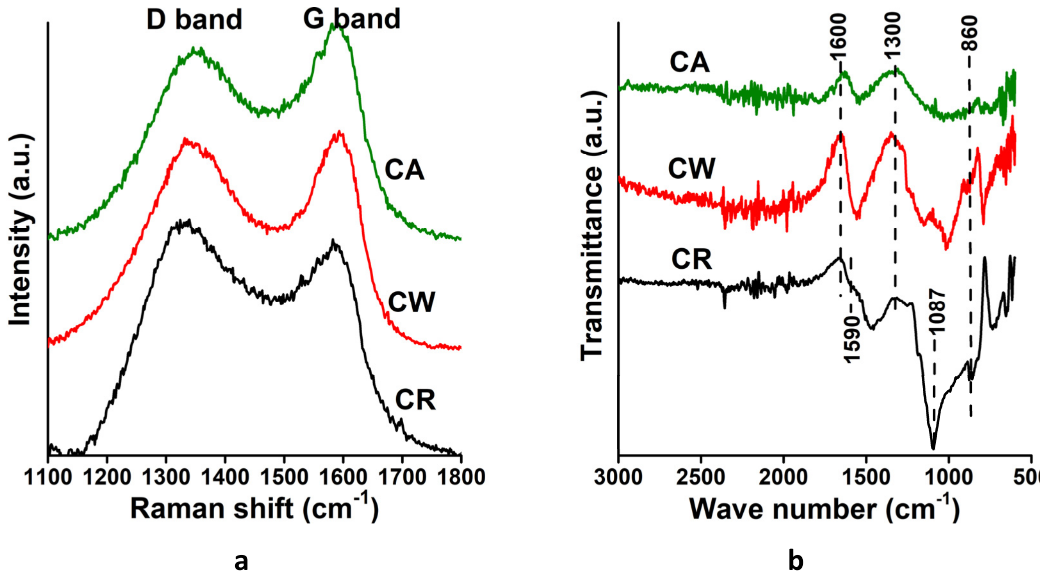


**Figure S4.** (**a**) Baseline corrected and normalized Raman spectra. ID/IG ratios are 1.06, 0.94, 0.95 for the CR, CW, and CA samples, respectively. (**b**) FTIR spectra of the precursors and CA. The range from 860 to 1300 $cm^{-1}$ corresponds to the C-O stretching vibrations [69–71], and the signal reduces significantly after washing and annealing steps because of the decrease in the oxygen content. A clear peak at 1087 $cm^{-1}$ appears in the spectrum for carbon raw (CR) and corresponds to S=O stretching [71]; this agrees with the presence of sulfur in the raw carbon, which is removed later with the acid washing. Also, the spectrum for the CR sample shows a small signal around 1590 $cm^{-1}$ which corresponds to N-H bending vibration [71], but it is not distinguishable in the two other samples. The signal at 1300–1600 $cm^{-1}$ corresponds to C=C from unoxidized $sp^2$ C-C bonds [69], O-H bending, C-OH stretching [70], and C-H bending vibrations [71]. This signal increases in the washed sample due to the acid washing step, but it becomes smaller in the annealed sample.

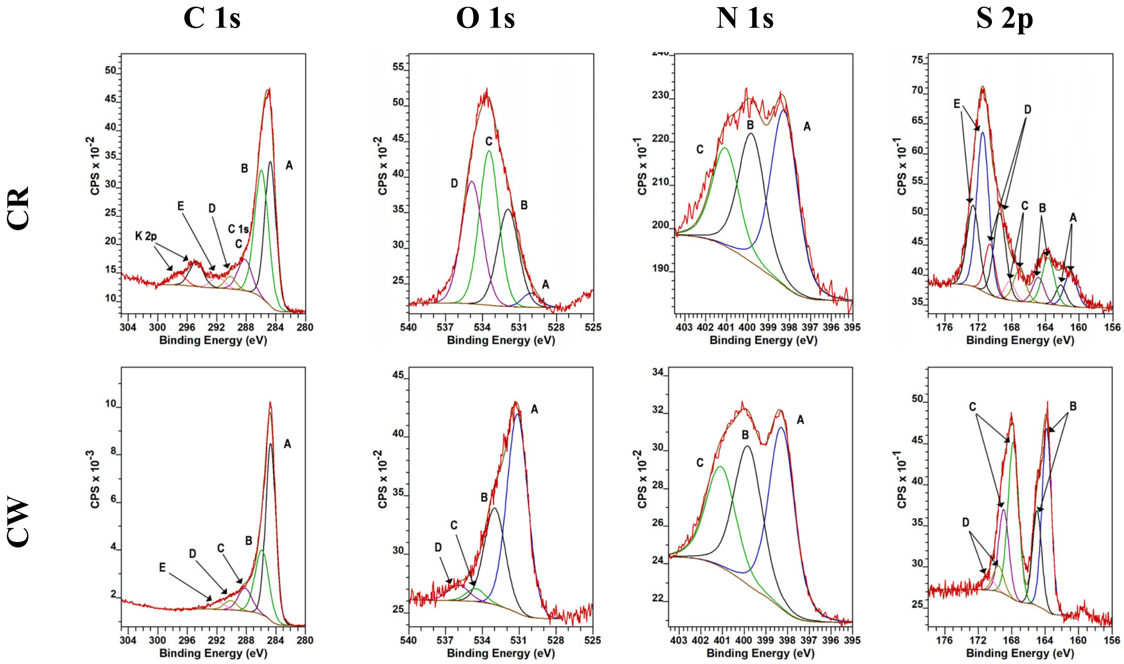


**Figure S5.** Deconvoluted regions of the XPS spectra of the porous carbon intermediates.

**Table S1.** Elemental compositions (atomic %) measured by EDS.

| Sample | C | O | N | S | K | Ca | Na |
|---|---|---|---|---|---|---|---|
| CR | 44.3 | 23.6 | 8.6 | 9.2 | 3.9 | 9.1 | 1.2 |
| CW | 73.4 | 6.3 | 17.5 | 2.8 | n.d. * | n.d. * | n.d. * |
| CA | 73.3 | 4.1 | 21.7 | 0.8 | n.d. * | n.d. * | n.d. * |

* n.d.: not detected

**Table S2.** Assignation of the chemical states in the C1s, O1s, N1s, and S2p 3/2 regions.

| Measured Energy (eV) | Label in Figures | Assignation | Component Ratios | | | Ref |
|---|---|---|---|---|---|---|
| | | | CR | CW | CA | |
| **C 1s** | | | | | | |
| 284.66–284.73 | A | graphitic C, disordered sp2 C-C | 39.6 | 55.5 | 59.5 | [18] |
| 285.86–285.93 | B | C-OH, epoxide | 42.8 | 27.8 | 23.7 | |
| 288.16–288.23 | C | C=O carbonyl | 10.6 | 9.8 | 9.1 | |
| 290.11–290.18 | D | O=C-OH carbonate | 3.4 | 3.7 | 4.1 | |
| 291.81–291.88 | E | π-π* shake up | 3.7 | 3.2 | 3.6 | |
| **O 1s** | | | | | | |
| 530.08–530.16 | A | O=C-OH, adsorbed O | 3.1 | 61.6 | 12.4 | [18] |
| 531.93–532.01 | B | quinine | 26.1 | 30.0 | 56.2 | |
| 533.48–533.56 | C | C-O-C, O=C-O* | 38.1 | 3.6 | 29.4 | |
| 534.88–534.96 | D | O related to S/N? | 32.8 | 4.8 | 2.1 | |
| **N 1s** | | | | | | |
| 398.25 | A | N-N pyridinic | 43.6 | 40.6 | 46.0 | [18] |
| 399.80 | B | pyrrolic N | 32.0 | 33.8 | 5.9 | |
| 400.95–401.06 | C | quaternary N, sp2 N in rings | 46.0 | 5.9 | 48.1 | |
| **S 2p** | | | | | | |
| 161.01 | A | thiol (-SH) | 9.6 | n.d. | n.d. | [72] |
| 163.69–163.88 | B | 2p3/2 (S1) and 2p1/2 (S2) positions of thiophene-S | 13.1 | 46.8 | 100 | [73,74] |
| 167.09–167.79 | C | -C-SO2- | 8.8 | 45.7 | n.d. | [74] |
| 169.46–169.58 | D | -C-SO4- | 23.8 | 7.5 | n.d. | |
| 171.47 | E | RO2-S-S-R, R-SO3H (in sulfonic acids) | 44.6 | n.d. | n.d. | [13] |

## References in supplementary material


69 Marcano, C. D.; Kosynkin, D. V.; Berlin, J. M.; Sinitskii, A.; Sun, Z.; Slesarev, A.; Alemany, L. B.; Lu, W.; Tour, J. M. Improved synthesis of graphene oxide. *ACS Nano* **2010**, *4*, 4806–4814.

70 Fernández-Merino, M. J.; Guardia, L.; Paredes, J. I.; Villar-Rodil, S.; Solís-Fernández, P.; Martínez-Alonso, A.; Tascón, J. M. D. RVitamin C is an ideal substitute for hydrazine in the reduction of graphene oxide suspensions. *J. Phys. Chem. C* **2010**, *114*, 6426–6432.

71. IR Spectrum Table & Chart. Available online: https://www.sigmaaldrich.com/technical-documents/articles/biology/ir-spectrum-table.html (accessed on 6 May 2021).

72. Su, Y.; Zhang, Y.; Zhuang, X.; Li, S.; Wu, D.; Zhang, F.; Feng, X. Low-temperature synthesis of nitrogen/sulfur co-doped three-dimensional graphene frameworks as efficient metal-free electrocatalyst for oxygen reduction reaction. *Carbon* **2013**, *62*, 296–301.
73. Seredych, M.; Khine, M.; Bandosz, T. Enhancement in Dibenzothiophene reactive adsorption from liquid fuel via incorporation of sulfur heteroatoms into the nanoporous carbon matrix. *ChemSusChem* **2011**, *4*, 139–147.
74. Yang, S.; Zhi, L.; Tang, K.; Feng, X.; Maier, J.; Mullen, K. Efficient synthesis of heteroatom (N or S)-doped graphene based on ultrathin graphene oxide-porous silica sheets for oxygen reduction reactions. *Adv. Funct. Mater.* **2012**, *22*, 3634–3640.
